# High FODMAP diet causes barrier loss via lipopolysaccharide-mediated mast cell activation

**DOI:** 10.1172/jci.insight.146529

**Published:** 2021-11-22

**Authors:** Prashant Singh, Gintautas Grabauskas, Shi-Yi Zhou, Jun Gao, Yawen Zhang, Chung Owyang

**Affiliations:** Divsion of Gastroenterology, Department of Medicine, University of Michigan, Ann Arbor, Michigan, USA.

**Keywords:** Gastroenterology, Mast cells

## Abstract

Fermentable oligosaccharides, disaccharides, monosaccharides, and polyols (FODMAPs) are carbohydrates thought to contribute to the symptoms of IBS. A diet in high in FODMAPs (HFM) induces gastrointestinal symptoms in patients with irritable bowel syndrome (IBS), and a diet low in FODMAPs (LFM) improves symptoms in up to 60% of patients with IBS. However, the mechanism by which FODMAPs affect IBS symptoms is unclear. We showed that mice fed on a HFM diet have mast cell activation and colonic barrier loss. Using mast cell–deficient mice with and without mast cell reconstitution, we showed that HFM-mediated colonic barrier loss is dependent on TLR4-dependent mast cell activation. In in vitro studies, we demonstrated that IBS fecal supernatant stimulates mast cells significantly more compared with fecal supernatant from healthy controls. This effect of IBS fecal supernatant on mast cell stimulation is ameliorated in the absence of the TLR4 receptor and after a LFM diet. We found that a LFM diet improves colonic barrier function and reduces mast cell activation while decreasing fecal LPS levels. Our findings indicate that a HFM diet causes mast cell activation via LPS, which in turn leads to colonic barrier loss, and a LFM diet reverses these pathophysiologic mucosal changes.

## Introduction

Irritable bowel syndrome (IBS) has a global prevalence of approximately 11%, with a significant negative impact on quality of life, work productivity, and economic burden on patients and healthcare (1–3). Up to 65% of patients with IBS report diet as triggers for their gastrointestinal symptoms (4–9). Studies have shown that fermentable oligosaccharides, disaccharides, monosaccharides, and polyols (FODMAPs) are thought to contribute to the symptoms of IBS, and a diet in high in FODMAPs (HFM) can induce characteristic IBS symptoms (10, 11). Moreover, fructo-oligosaccharides and fructose (crucial components of FODAMPs) have been shown to generate IBS symptoms in a dose-dependent manner (10, 11). Conversely, a diet low in FODMAPs (LFM) has been shown to improve IBS symptoms in several randomized controlled trials (12). Based on predominant altered bowel habit, IBS can be divided into constipation-predominant IBS, diarrhea-predominant IBS (IBS-D), and IBS with mixed subtype (3). In this study, patients with IBS-D were selected for our investigation because the data on the efficacy of LFM diet in IBS is strongest for the IBS-D subtype, and randomized controlled trials including only patients with IBS-D subtype have shown significant improvement in abdominal pain, bloating, stool consistency, stool frequency, and fecal urgency (4, 13, 14). A recent meta-analysis pooled the data from 6 randomized controlled trials in which the majority of patients were of IBS-D subtype, showing a LFM diet was associated with significantly higher reduction in IBS symptom severity compared with control intervention (14). Based on these observations, a LFM diet is often recommended as first-line therapy for patients with IBS-D (15). However, the mechanisms by which FODMAPs cause IBS-D symptoms are not well understood (16, 17).

Several studies, including a recent meta-analysis, have shown that mast cells are increased in number in the colonic mucosa of a subset of patients with IBS-D (18). Moreover, a subset of patients with IBS-D has increased mast cell degranulation and elevated levels of mast cell mediators such as histamine, prostaglandin E2 (PGE2), and tryptase in the colonic mucosa (19). These bioactive molecules released via mast cell activation, including PGE2, histamine, tryptase, and cytokines, have been shown to cause epithelial barrier loss in in vitro and/or in vivo models (20–24). Interestingly, a subset of patients with IBS-D also has altered colonic barrier function (25–27) and factors known to affect gut barrier function such as stress are also known to exacerbate IBS-D symptoms (28). Furthermore, the magnitude of barrier dysfunction and mast cell activation has been shown to correlate with IBS-D symptom severity (25, 26, 29, 30). However, the factors leading to mast cell activation in IBS-D are poorly understood.

As a key mucosal immune cell, mast cells have the ability to respond directly to dietary antigens (e.g., via crosslinking of antigen-specific IgE to FcεRI) and indirectly to diet-induced gut dysbiosis (e.g., via pattern recognition receptors such as TLRs or CD48) (31, 32). Based on these observations, mast cells have been speculated to have a key role in diet and dysbiosis-mediated events leading to IBS symptoms; however, this has been poorly investigated (32). To date, no study has investigated the role of mast cells in FODMAP-mediated IBS-D pathogenesis.

Diet is known to modulate the gut microbiome, and studies have shown that gram-negative phyla *Verrucomicrobia* (family *Akkermansiaceae*) and *proteobacteria* (family *Enterobacteriaceae*) increase in rodents fed on fructo-oligosaccharides and fructose, respectively (both of which are crucial components of FODMAPs) (33–36). Furthermore, we recently showed that this HFM-mediated gram-negative microbial shift leads to increased fecal LPS levels in rodent models (LPS is a key component of gram-negative bacterial outer membrane). We further showed that these luminal changes were associated with colonic barrier dysfunction and recruitment of mast cells to colonic mucosa (33). Normally, LPS cannot penetrate across the healthy colonic epithelium. However, animal studies have shown that, in the presence of bacterial dysbiosis and/or dietary manipulation, LPS can migrate across the colonic epithelium via a transcellular route (37–41). Once LPS is on the basolateral side, it can activate TLR4 receptors on various immune cells, including mast cells, to release cytokines such as TNF-α, IL-1β, and IL-6, as well as proteases such as tryptase (42–44). We previously showed that levels of fecal or luminal LPS is elevated in IBS-D, which is also consistent with findings of elevated serum LPS in these patients (33, 45). However, the functional significance of elevated fecal LPS in IBS-D — i.e., its interaction with colonic epithelial or immune cells in IBS-D — has yet not been studied. In this study, we hypothesize that mast cells are critical for HFM diet–mediated colonic epithelial barrier dysfunction and that luminal LPS plays a key role in HFM diet–mediated mast cell activation.

To test this hypothesis, we conducted experiments in WT and mast cell–deficient mice to determine if mast cells are essential for FODMAP-mediated colonic epithelial barrier dysfunction. Translating these findings in human, in a separate clinical study, we examined the effects of a LFM diet on colonic epithelial barrier dysfunction and mast cell activation seen in patients with IBS-D. Finally, we examined the effects of fecal supernatants from patients with IBS-D (before and after a LFM diet) on mast cell activation using BM-derived mast cells (BMMCs) of WT and TLR-KO (*tlr*^–/–^) mice.

## Results

### HFM causes colonic barrier loss and mast cell activation.

**Figure 1:**
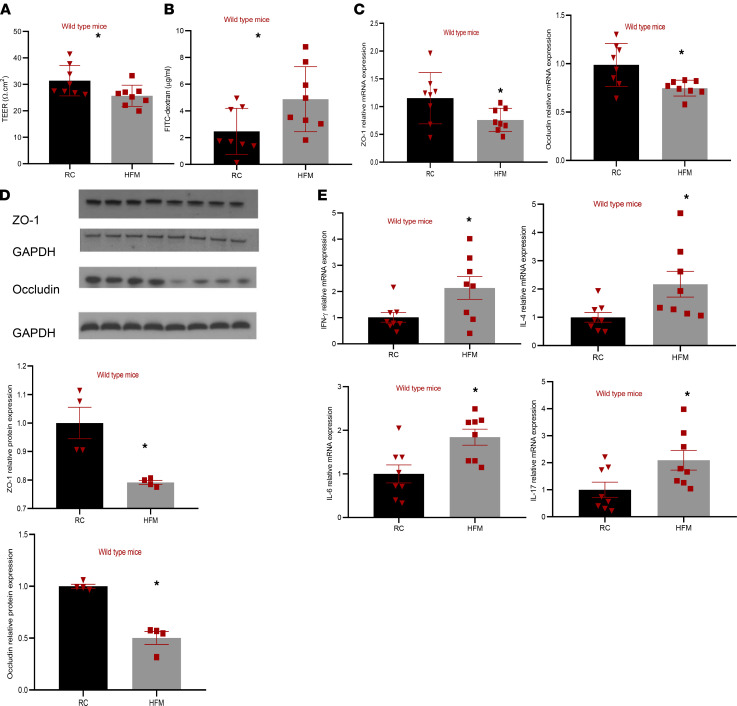
HFM diet causes colonic barrier loss in WT mice. Adult male C57BL/6 mice were randomized for 2 weeks to a HFM diet versus regular chow (RC). (**A** and **B**) A HFM diet caused barrier loss, as evidenced by reduced transepithelial electrical resistance of mice colonic tissue and an increase in plasma concentration of 4 KDa FITC–dextran after oral gavage. (**C**–**E**) A HFM diet also caused a significant decrease in relative mRNA expression of tight junction proteins (ZO-1 and Occludin), a significant reduction in relative protein expression of tight junction proteins (ZO-1 and Occludin), and a significant increase in gene expression of inflammatory markers such as IFN-γ (*n* = 8/group). *P < 0.05 for each using unpaired *t* test.

HFM-fed mice had significantly lower transepithelial electrical resistance (TEER) compared with mice fed on regular chow (RC) (25.7 ± 4 Ω.cm^2^ versus 31.4 ± 5.7 Ω.cm^2^, *P* = 0.037) (Figure 1A). Consistent with reduced TEER, HFM-fed mice also showed an increase in in vivo permeability with significantly higher plasma FITC-dextran concentration after oral gavage (*P* = 0.038; Figure 1B). We also found that gene expression of ZO-1 and Occludin were reduced by 33.9% ± 15.5% (*P* = 0.04) and 24.3% ± 8.1% (*P* = 0.019), respectively, in HFM-fed mice (Figure 1C). This was further confirmed by reduced ZO-1 and Occludin protein expression in colonic mucosa of HFM-fed mice compared with RC-fed mice (*P* < 0.05 for both; Figure 1D). There was no difference in gene expression of other tight junction proteins such as JAM-A and Claudin 1 (Supplemental Figure 1; supplemental material available online with this article; https://doi.org/10.1172/jci.insight.146529DS1). There was also increased gene expression of IFN-γ, IL-4, IL-6, and IL-17a (Figure 1E), but the levels of other cytokines such as IL-1, IL-10, and TNF-α were similar in the 2 groups (Supplemental Figure 1).

**Figure 2:**
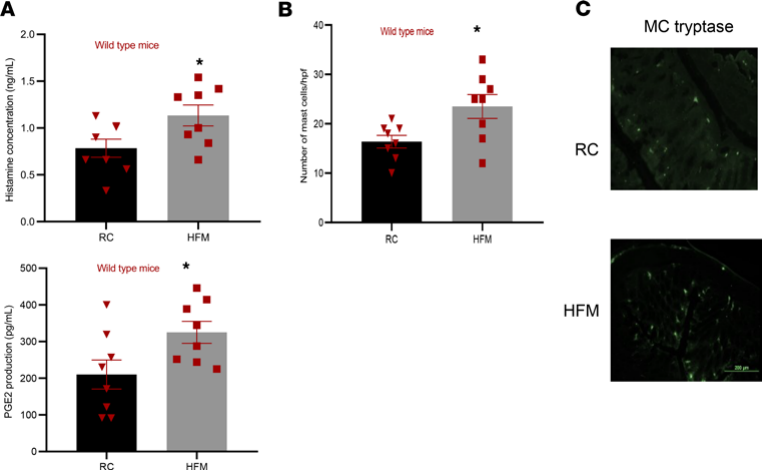
HFM diet causes colonic mast cell recruitment and activation in WT mice. Adult male C57BL/6 mice were randomized for 2 weeks to a HFM diet versus regular chow (RC). (**A**) A HFM diet caused mast cell activation as evidenced by increased mucosal levels of mast cell mediators such as histamine and prostaglandin E2 (PGE2). (**B**) A HFM diet also cause mast cell recruitment to colonic mucosa (**C**) Immunohistochemical studies show mast cell tryptase immunoreactivity in colonic mucosa of HFM-fed and RC-fed WT mice (*n* = 8/group). *P < 0.05 for each using unpaired *t* test. Scale bar: 200 μm.

HFM-induced barrier dysfunction was associated with significant increases in mucosal levels of histamine and PGE2, markers of mast cell activation (*P* = 0.03 and 0.04, respectively; Figure 2A). This was accompanied with mast cell recruitment to colonic mucosa in HFM-fed mice compared with RC-fed mice (*P* = 0.007; Figure 2, B and C).

### Mast cells are critical for HFM-mediated colonic barrier loss.

**Figure 3:**
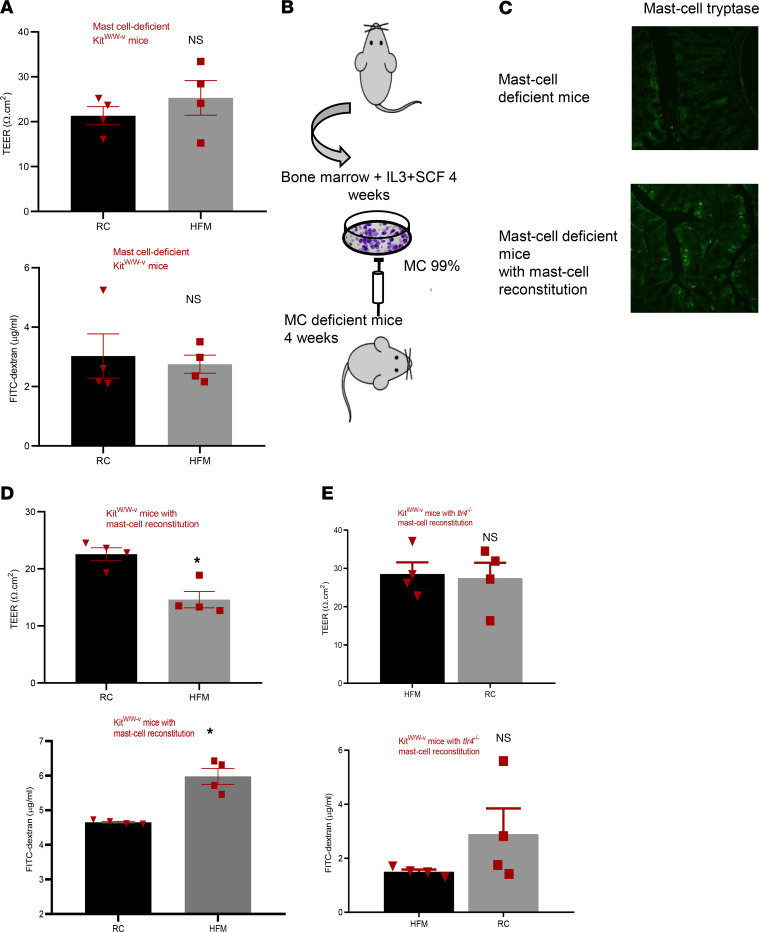
HFM diet–induced colonic barrier loss is mediated via mast cell activation. Mast cell–deficient Kit^W/W–v^ (MCD) mice were randomized for 2 weeks to a HFM diet versus regular chow (RC) for 2 weeks. (**A**) A 2-week HFM diet did not cause barrier loss in MCD mice - trans-epithelial electrical resistance (TEER) of mice colonic tissue, plasma concentration of 4 KDa FITC–dextran after oral gavage were similar in HFM-fed versus RC-fed MCD mice (*n* = 4/group). (**B**) Experimental design of BM-derived mast cell reconstitution in MCD. Mast cells (MCs) were derived from BM of WT mice and cultured with IL-3/stem cell factor for 4 weeks. Reconstitution occurred within 4 weeks after transfer of these BM-derived MCs via tail-vein injection. (**C**) IHC studies show MC tryptase immunoreactivity in colonic mucosa of MCD mice and reconstituted MCD (MCR) mice 4 weeks after injection. (**D**) Mast cell reconstitution with WT mice in MCD mice restored the ability of a HFM diet to induce colonic barrier loss, whereas RC did not have any effect on barrier function of MCR mice. This is reflected in significantly lower colonic TEER and significantly higher plasma concentration of 4 KDa-FITC-dextran in HFM-fed MCR mice compared with RC-fed MCR mice (*n* = 4/group). (**E**) Mast cell reconstitution of mast cell deficient mice with *tlr4^–/–^* mast cells did not experience any effect on barrier function related to HFM compared with their RC-fed counterparts. *P < 0.05, determined using unpaired *t* test.

To understand the role of mast cells in HFM-induced colonic barrier loss, we investigated the effect of a HFM diet on mast cell deficient (Kit^W/W–v^) mice. We found that TEER and plasma FITC-dextran concentration after oral gavage were not significantly different between the 2 groups in mast cell–deficient Kit^W/W–v^ mice (Figure 3A). Similarly, gene expression of tight junction proteins (ZO-1, Occludin, JAM-A, or Claudin 1) or inflammatory markers (IFN-γ, IL-1, and TNF-α) were not significantly different between mast cell–deficient Kit^W/W–v^ mice randomized to RC versus HFM groups (Supplemental Figure 2).

Mast cell–deficient Kit^W/W–v^ mice were reconstituted with mast cells from WT mice as described below (Figure 3, B and C). Six weeks after mast cell reconstitution, these mice were randomized to HFM versus RC for 2 weeks. TEER was significantly lower in the HFM group (22.6 ± 2.3 Ω.cm^2^) compared with the RC group (14.6 ± 2.9 Ω.cm^2^, *P* = 0.005). Consistent with reduced TEER, plasma FITC-dextran concentration after oral gavage was also significantly higher in the mast cell–reconstituted Kit^W/W–v^ mice fed on a HFM diet compared with RC group (*P* = 0.001) (Figure 3D).

In separate studies, mast cell–deficient Kit^W/W–v^ mice were reconstituted with mast cells from *tlr4^–/–^* mice, and 6 weeks after reconstitution, these mice were randomized to HFM versus RC for 2 weeks. In contrast to the findings seen in mast cell reconstitution with mast cells from WT mice, when mast cells from *tlr4*^–/–^ mice were used for reconstitution, there was no significant difference between colonic TEER in HFM group (28.6 ± 3 Ω.cm^2^) and the RC group (27.5 ± 4 Ω.cm^2^, *P* = 0.84). We also did not observe any significant difference in plasma FITC concentration between the 2 groups when *tlr4*^–/–^ mast cells were used for reconstitution (Figure 3E).

### FODMAP-driven mast cell activation in IBS-D is mediated via luminal LPS.

**Figure 4:**
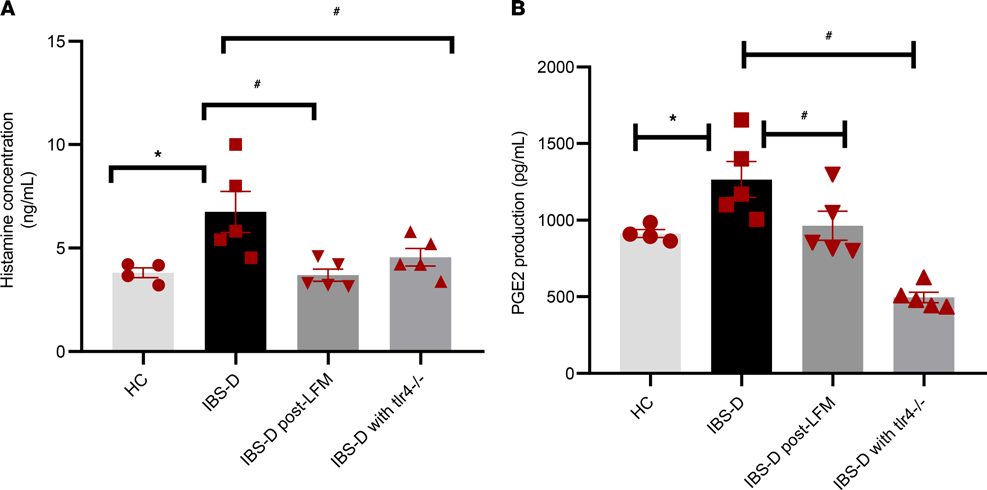
FODMAP-induced mast cell activation is mediated via LPS. In total, 1 × 10^5^ BM mast cells/well were plated in a 24-well plate and treated with fecal supernatants from 4 healthy controls (HC) and 5 patients with IBS-D before and LFM (each responded clinically to LFM). Baseline IBS-D fecal supernatants (pre-LFM) was also applied to BMMCs derived from *tlr4^–/–^* mice. (**A** and **B**) Culture supernatants were collected after 1 and 5 hours to measure histamine (**A**) and Prostaglandin E2 (PGE2) (**B**) concentration, respectively. Baseline (pre-LFM) IBS-D fecal supernatants increased histamine and PGE2 production compared with HC. Histamine and PGE2 concentration reduced after LFM and remained low when baseline IBS-D supernatants were applied to *tlr4^–/–^*BMMCs (*n* = 5/group).*P < 0.05versus HC, ^#^*P* < 0.05 versus IBS-D, determined by 1-way ANOVA followed by Dunnett’s multiple-comparison test.

We also performed in vitro studies using pre- and post-LFM fecal supernatants from same patients with IBS-D (see below) obtained before and after 4 weeks of a LFM diet, as well as fecal supernatants from healthy controls. IBS-D fecal supernatants stimulated WT mice–derived BMMCs to produce significantly higher levels of histamine and PGE2 compared with healthy controls (*P* = 0.01 and 0.03, respectively) (Figure 4, A and B). When comparing the effects of baseline (pre-LFM) IBS-D fecal supernatants on WT and *tlr4*^–/–^ BMMCs (same fecal supernatant samples added to both groups), histamine and PGE2 production remained significantly lower in *tlr4*^–/–^ BMMCs compared with WT BMMCs (*P* = 0.04 and 0.001, respectively; Figure 4, A and B). Compared with stimulation of WT BMMCs with baseline (pre-LFM) fecal supernatants, post-LFM fecal supernatants from the same patients did not stimulate production of histamine and PGE2 production from WT BMMCs (*P* = 0.047 and *P* = 0.006, respectively) (Figure 4, A and B).

### LFM improves colonic barrier function and mast cell activation in patients with IBS-D.

We studied the colonic barrier function and mast cell activation in 6 patients with IBS-D who responded to a 4-week LFM diet. patients with IBS-D had moderate to severe IBS severity at baseline, as evidenced by mean IBS severity scoring system (IBS-SSS) score of 312 (± 56.9). After a LFM diet, all 6 patients had symptomatic response with decreases in IBS-SSS scores by ≥ 50 points. The mean IBS-SSS score after a 4-week LFM diet was 65.3 (± 61.6) (*P* < 0.001). Mean patient-reported outcomes measurement information system (PROMIS) abdominal pain T-score (standard scores with a mean of 50 and standard deviation of 10 in US general population) improved from 62.5 at baseline to 38.8 at the end of 4 weeks of a LFM diet (*P* = 0.002). Similarly, mean PROMIS diarrhea T-score also improved from 59.6 at baseline to 44.4 at the end of 4 weeks of a LFM diet (*P* = 0.02). With 4-week LFM dietary intervention, mean daily stool consistency improved from Bristol stool form scale (BSFS) 5.4 before LFM to BSFS 4.1 after 4 weeks of LFM (*P* = 0.009). There was improvement in mean daily stool frequency from 3.1/week before LFM to 2.5/week after 4 weeks of LFM, but this was not significant (*P* = 0.2)

**Figure 5:**
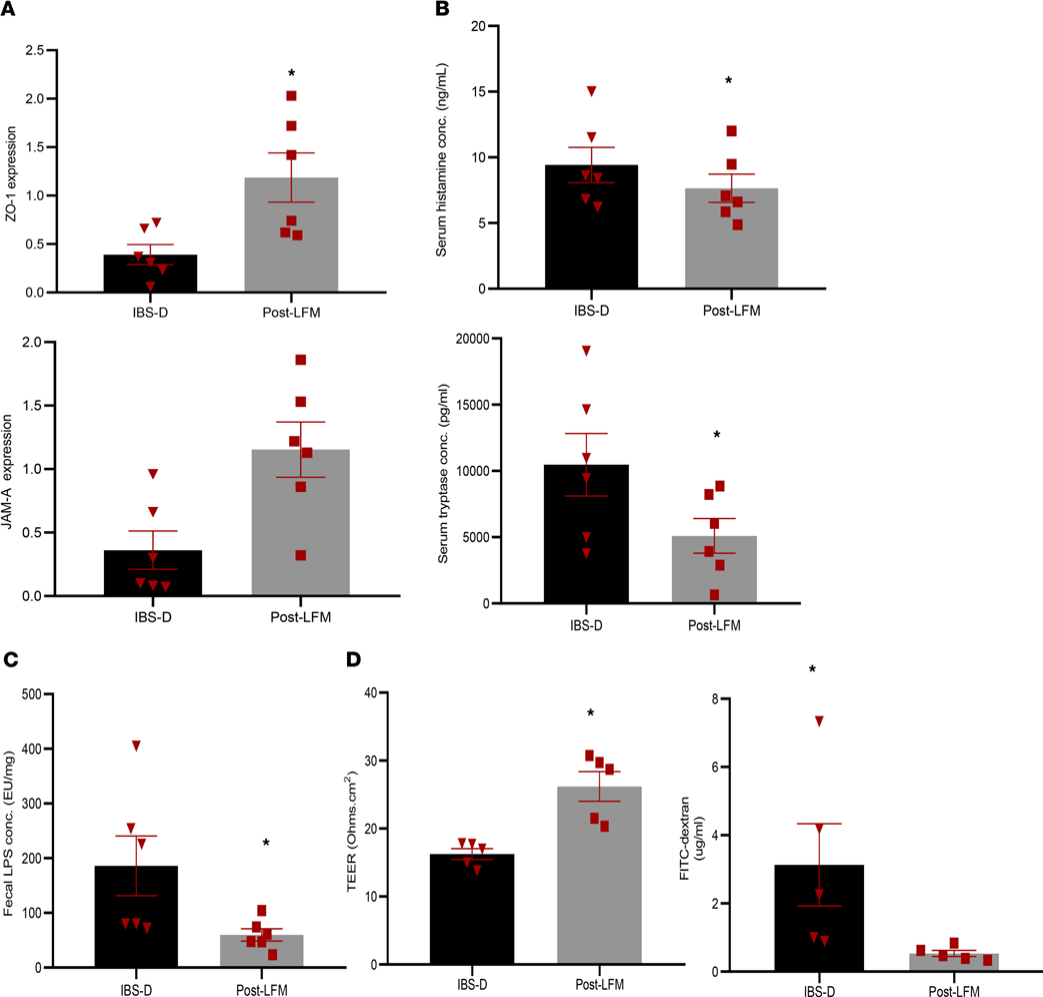
LFM diet improved barrier function and mast cell activation and reduced fecal LPS levels in patients with IBS-D. Six patients with IBS-D were provided dietitian prepared LFM diet for 4 weeks, and all patients had a clinical response. Colonic biopsies and serum and fecal specimens were obtained from IBS-D before and after a 4-week LFM diet. (**A**) A LFM diet improved tight junction dysfunction seen in patients with IBS-D and significantly increased the gene expression of tight junction proteins ZO-1 and JAM-A. (**B** and **C**) This was accompanied with significant reduction in serum markers of mast cell activation (mast cell tryptase and histamine) and a decrease in fecal LPS levels (*n* = 6/group). (**D**) In a separate experiment, pre-LFM (baseline) and post-LFM (after 4-week LFM diet) IBS-D fecal supernatants (200 μL) from these patients (*n* = 5) were administered intracolonically every day for 5 days to naive mice. After 5-day intracolonic fecal supernatant administration, mice injected with post-LFM fecal supernatant had higher TEER and lower plasma FITC concentration compared with pre-LFM IBS fecal supernatant mice, suggesting that pre-LFM IBS fecal supernatant causes barrier loss, which is reversed by LFM. *P < 0.05, using paired *t* test (**A**–**C**) and unpaired *t* test (**D**).

After a 4-week LFM diet, there was a significant improvement in the mean levels of gene expression of tight junction proteins JAM-A (0.36 versus 1.15, *P* = 0.023) and ZO-1 (0.39 versus 1.19, *P* = 0.031) (Figure 5A). There was no significant change in the levels of other tight junction proteins (Occludin and Claudin 1) (data not shown). This was accompanied with significant reduction in serum levels of histamine and mast cell tryptase post-LFM diet (*P* = 0.033 and *P* = 0.049, respectively) (Figure 5B). However, there was no change in the fecal levels of histamine before and after a LFM diet for 4 weeks, suggesting histamine was not bacterial or dietary in origin. We also found that a LFM diet for 4 weeks significantly reduced the mean fecal LPS concentration in these LFM-responsive patients with IBS-D (186 ± 133.9 EU/μg versus 59.7 ± 26.7 EU/ μg, *P* = 0.045) (Figure 5C).

To further validate our findings seen in human subjects, we performed an in vivo study where 200 μL fecal supernatants from patients with IBS-D (before and after LFM) from the same patients were administered intracolonically to naive WT mice for 5 days. TEER was significantly lower in mice injected with baseline (pre-LFM) IBS fecal supernatant compared with those injected with post-LFM IBS fecal supernatant from the same patients (16.2 ± 1.8 Ω.cm^2^ versus 26.2 ± 4.9 Ω.cm^2^, *P* = 0.003) (Figure 5D). Similarly, plasma FITC concentration was significantly higher in mice injected with baseline (pre-LFM) IBS fecal supernatant–treated mice compared with post-LFM IBS fecal supernatant–treated mice (*P* = 0.008) (Figure 5D).

## Discussion

Our study shows that a HFM diet causes colonic mast cell activation and barrier dysfunction in rodent models and that TLR4-dependent mast cell activation is critical for this FODMAP-induced colonic barrier dysfunction. Translating these findings in patients with IBS-D, we found that a LFM diet improves colonic barrier function and reduces mast cell activation. We also found that luminal LPS plays a key role in mediating FODMAP-driven mast cell activation in a subset of patients with IBS-D.

FODMAPs are known to cause gastrointestinal symptoms in patients with IBS-D in a dose-dependent manner, and a LFM diet alleviates IBS-D symptoms in up to 60% of patients with IBS-D (10–13). However, the mechanisms by which FODMAPs cause IBS-D symptoms are not well understood. FODMAPs are poorly absorbed by the small intestine and fermented by bacteria in the colon to produce gas and osmotically active carbohydrates; these events act in concert to cause bloating and diarrhea. FODMAPs may also serve as nutrients for colonic bacteria and promote osmosis (31). We previously showed that a HFM diet increases fecal LPS levels by causing gram-negative dysbiosis in rodent models. We and others have shown that gram-negative phyla *Verrucomicrobia* (family *Akkermansiaceae*) and *proteobacteria* (family *Enterobacteriaceae*) increase in rodents fed on fructo-oligosaccharides and fructose, respectively (both of which are crucial components of FODMAPs) (33–36). Normally, LPS cannot penetrate across the healthy colonic epithelium. However, animal studies have shown that, in the presence of bacterial dysbiosis and/or dietary manipulation, LPS can migrate across the colonic epithelium via a transcellular route (37–41). Once on the basolateral side, LPS can activate mast cells via a TLR4-dependent pathway to stimulate the release of several bioactive molecules. We previously showed that HFM-mediated colonic barrier loss is normalized in in vivo rodent models in the presence of an LPS antagonist (33). However, it was unclear if this barrier loss was due to the direct effect of fecal LPS on colonic epithelial cell or via LPS-mediated immune cell activation. The current study suggests that the effect of LPS on FODMAP-related barrier loss is mediated via LPS-driven mast cell activation.

In this study, using rodent models, we have shown that a HFM diet leads to colonic barrier loss and mast cell activation, key pathophysiologic findings seen in a subset of patients with IBS-D. Moreover, a LFM diet significantly increases colonic mRNA expression of tight junction proteins (JAM-A and ZO-1) in patients with IBS-D who were LFM-diet responders. LFM also reduced mast cell activation in patients with IBS-D reflected by decreased serum levels of mast cell tryptase and histamine. This is consistent with a previous study reporting decreased urinary levels of histamine with LFM in patients with IBS-D (46). Of note, downregulation of JAM-A and ZO-1 expression in the gut mucosa, as well as an increase in systemic and/or mucosal levels of mast cell activation products (such as tryptase and histamine), has been reported in patients with IBS-D (19, 25, 47–51). Interestingly, the magnitude of barrier loss and mast cell activation has been shown to correlate with symptom severity in IBS-D (25, 29, 30, 48, 52). Future studies should explore if there is a differential effect of a LFM diet on barrier function and mast cell activation among IBS-D responders and nonresponders. We did not have nonresponders among the 6 patients with IBS-D we recruited.

To our knowledge, ours is the first study reporting a HFM diet–mediated colonic mast cell activation and establishes mast cells as key mediators of colonic barrier loss caused by a HFM diet. This builds upon the previous observations from our group and others that a HFM diet causes recruitment of mast cells to colon mucosa in rodent models (33, 53, 54). However, these studies did not study mast cell activation or physiological consequences of HFM-mediated mast cell recruitment. Mast cell activation releases several bioactive molecules, including histamine, tryptase, and PGE2, which have all been shown to cause epithelial barrier loss and increase paracellular permeability in in vitro models (20–24). Moreover, several clinical studies have shown a positive correlation between severity of mast cell activation and magnitude of barrier loss in patients with IBS-D (52, 55, 56). Finally, mast cell stabilization has been shown to improve barrier function in ex vivo studies from patients with IBS-D (57).

In our study, with the help of WT and *tlr4*^–/–^ mast cell reconstitution in mast cell–deficient mice, we were able to show that TLR4 receptor on mast cells is critical for this HFM-mediated colonic barrier loss, as HFM-mediated colonic barrier loss did not occur in the absence of mast cells or in the presence of mast cells that lacked a TLR4 receptor. This is consistent with our previous observation that HFM-mediated barrier loss is reversed in in vivo rodent models in the presence of LPS antagonist (33).

We found that IBS-D fecal supernatants stimulated mast cells to a significantly higher degree compared with fecal supernatants from healthy controls. IBS-D fecal supernatant–mediated mast cell stimulation was significantly reduced after a LFM diet or when fecal supernatants were applied to *tlr4^–/–^* mast cells. We also showed that a LFM diet reduces fecal LPS levels. Taken together, these findings suggest that mast cell activation in a subset of patients with IBS-D is mediated via LPS and that a LFM diet reverses mast cell activation in these patients with IBS-D. Although mast cell activation has been shown in several clinical IBS-D studies, etiology for mast cell activation in IBS-D remains poorly understood. We provide the first evidence to our knowledge that HFM-induced microbial dysbiosis is associated with mast cell activation in a subset of patients with IBS-D.

Our study has several limitations. Firstly, we did not have gene expression data on tight junction proteins and inflammatory cytokines in mast-cell–reconstituted mice. Secondly, our sample size for human studies was small (*n* = 6), and these findings need to be confirmed in a larger clinical trial. Thirdly, we did not have data on the microbiome composition of patients with IBS-D before and after LFM, and future studies should investigate the bacterial source of this HFM-mediated increase in fecal LPS levels. Lastly, for rodent experiments, whole colonic tissue — not mucosa — was used for the assessment of barrier function and mast cell activation. Despite these limitations, our study has several strengths. Our data posits FODMAP-mediated dysbiosis–derived LPS as a key mediator for mast cell activation in a subset of patients with IBS-D and is the first study to our knowledge to show that the mast cell activation is critical for FODMAP-mediated barrier loss. Taken together, findings from the current study and our previous work challenges the current dogma that the beneficial effect of a LFM diet on symptom resolution in IBS-D is solely related to reduced luminal distention secondary to decreased fermentation of carbohydrates (33). The molecular mechanisms of how HFM-mediated mast cell activation leads to colonic epithelial barrier loss and future studies should investigate this in more detail.

In conclusion, we have shown that a HFM diet causes TLR4-dependent mast cell activation, which in turn leads to colonic barrier loss in rodent models. Similar observations were made in patients with IBS-D who show improvement in colonic barrier function, mast cell activation, and fecal LPS levels with a LFM diet. In vitro studies using paired fecal supernatants samples from IBS-D before and after LFM suggests that HFM-mediated mast cell activation is due to luminal LPS.

## Methods

### Animals and diet.

Adult male C57BL/6 mice (*n* = 8/group, Charles River Laboratories) and mast cell–deficient Kit^W/W–v^ mice (*n* = 4/group, The Jackson Laboratory) were housed 4 per cage in a controlled environment (12-hour daylight cycle, lights off at 18:00) with free access to food and water (allowed to eat ad libitum). Mice were randomized into 2 groups and, for 14 days, were fed a HFM diet or RC. The composition of the HFM diet was based on a human clinical study: 10% w/w FODMAPs, comprising 3.6% w/w fructose, 3.6% w/w lactose, and 3% w/w fructo-oligosaccharides (D19102503, Research Diets) (10). Each gram of HFM diet and RC diet (D19102504, Research Diets) provided 3.83 Kcal and 3.78 Kcal, respectively, with 16% of calories provided by fat, 64% of calories provided by carbohydrate, and 20% of calories provided by protein in each group. Percent total fiber by weight was 9.3% in both groups, with cellulose providing the entirety of the fiber in the RC group and with cellulose, along with FODMAPs, providing the fiber content in the HFM group.

### Mast cell reconstitution.

Selective reconstitution of mast cells in mast cell–deficient Kit^W/W–v^ mice (The Jackson Laboratory) was conducted according to the method described by Rijnierse et al. (58). BMMCs were obtained from WT (C57BL/6) and *tlr4*^–/–^ mice (C3H/HeJ, The Jackson Laboratory). BM was aseptically flushed from femurs and cultured for 4 weeks. Mast cell–deficient Kit^W/W–v^ mice were injected via the tail vein with 5 × 10^6^ cultured mast cells, and the recipients (*n* = 4/group) were randomized to HFM diet or RC 4 weeks later for a duration of 2 weeks.

### TEER.

The ex vivo intestinal barrier function was assessed by measurement of TEER as reported previously (33). TEER, along with dextran flux (described below), is a quantitative and sensitive measure of epithelial barrier integrity and paracellular permeability. Intestinal tissue from the proximal colon was used for these experiments, since the majority of the FODMAPs are poorly absorbed and readily metabolized by colonic microbiome in proximal colon (59). Intestinal segments were opened along the mesenteric border, washed in phosphate-buffered saline (PBS), and cut into 5 × 7 mm pieces. Tissues were washed twice in sterilized PBS and transferred to Petri dishes containing DMEM culture medium. After a 30-minute incubation at 37°C and pH stabilization, the TEER was measured using the micro-Snapwell system with an Endohm SNAP electrode attached to an EVOM2 epithelial volt-ohm meter (World Precision Instruments) and expressed in Ω.cm^2^.

### In vivo dextran flux measurement.

In vivo permeability measurement was modified from previously described methods based on gut permeability to 4 kDa FITC–dextran. Mice were fasted for 6 hours and gavaged with 4 kDa FITC–dextran (0.5 mL, 100 mg/mL). After 1 hour, whole blood was collected using a retro-orbital approach. Plasma was diluted in an equal volume of PBS (pH 7.4), and the FITC-dextran concentration was determined with a Synergy 2 microplate reader (BioTek), with serial dilutions of FITC-dextran used as a standard curve.

### Quantitative PCR for tight junction proteins and inflammatory cytokines.

Total RNA was extracted from proximal colon tissue samples using Trizol reagent (Invitrogen), according to the manufacturer’s instructions. cDNA was synthesized using iScript cDNA synthesis kit (Bio-Rad). Quantitative PCR (qPCR) for tight junction proteins, inflammatory cytokines, and GAPDH was performed with a CFX Connect Real-Time PCR Detection System (Bio-Rad) using SYBR Green detection. Primers used for qPCR, GAPDH, ZO-1, Occludin, JAM-A, Claudin 1, IL-1β, TNF-α, and IFN-γ were obtained from Qiagen. The PCR conditions were as follows: 1 cycle at 95°C for 10 minutes, followed by 40 two-temperature cycles at 95°C for 15 seconds and 60°C for 60 seconds. PCR amplifications were performed in a total volume of 25 μL, containing iQSYBR Green supermix (Bio-Rad). Cytokine and tight junction protein transcript levels were normalized to that of GADPH, and relative gene expression was expressed as the fold change (2^−ΔΔCt^) relative to expression in the control samples.

### Western blot analysis.

Proteins were extracted from the proximal colon tissues and analyzed on Ready Gel Tris-HCl (Bio-Rad). The tissues were homogenized in RIPA buffer (1% IGEPAL, 0.5% sodium deoxycholate, and 0.1% SDS in Tris-buffered saline solution [pH 7.4]), supplemented with protease inhibitor cocktail (Sigma-Aldrich). The homogenate was centrifuged at 14,000*g* for 10 minutes at 4°C. Sample lysates were denatured at 95°C for 5 minutes in the presence of 4× LDS sample loading buffer (Invitrogen) and 5% β-mercaptoethanol (Bio-Rad). Equal amounts of protein (30 μg) were separated by 4%–20% Ready Gel Tris-HCl gels (Bio-Rad), transferred to polyvinylidene difluoride membranes, and blocked with StartingBlockT20 blocking buffer (Thermo Fisher Scientific) for 60 minutes at room temperature. Membranes were incubated with rat anti–ZO-1 monoclonal antibody (MABT 11, Sigma-Aldrich) and rabbit recombinant monoclonal anti-OCLN antibody (ab167161, Abcam) at 1:400 dilution at 4°C overnight, and they were then washed in Tris-buffered saline for 1 hour. The membranes were then probed with peroxidase-conjugated secondary antibodies at 1:8000 dilution for 1 hour at room temperature, and the bands were visualized by electrochemiluminescence (ECL, Thermo Fisher Scientific). Signals were quantified using ImageJ (NIH) and normalized to controls.

### Mucosal histamine and PGE2 measurement.

Animal colon specimens were collected as described previously (19). The tissues were rapidly immersed in hard plastic tubes containing 1 mL Dulbecco’s PBS media and continuously oxygenated (95% O_2_/5% CO_2_) at 37°C. After a 30-minute incubation, the bathing solution was removed, filtrated, and stored at –80°C. At the end of the experiment, biopsies were weighed. ELISA assays of PGE2 (500141, Cayman Chemical) and histamine (589651, Cayman Chemical) were performed according to the instructions provided by the manufacturer. 

### Mast cell staining.

Colon tissue samples were collected from proximal colon after sacrificing mice and fixed in 4% paraformaldehyde. Following this, they were embedded in paraffin, sectioned at 5 μm thickness, and stained for mast cells. For immunohistochemical staining, colonic biopsy sections were incubated with rabbit monoclonal recombinant anti–mast cell tryptase (ab-134932, Abcam). Mast cells were counted at a magnification of ×400 in 8 different areas above the muscularis mucosae of each section using a micrometer grid and expressed as the number of cells/hpf.

### Human studies.

Six patients with IBS-D (4 females and 2 males) with mean (± SD) age of 30.3 (± 4.84) were recruited from the outpatient gastroenterology clinic. IBS-D subjects were chosen for our study, since most of the studies on a LFM diet have focused on this subtype of IBS and the data on efficacy of a LFM diet is most robust for IBS-D subjects. After a 7-day screening period (day –7 to 0), patients went on a 4-week LFM diet (day 0 to 28). To ensure dietary compliance, patients were counseled about LFM diet at the beginning of the study, and they received daily LFM meals (3 meals and 2 snacks) for the duration of the study. IBS symptom severity was measured using the IBS-SSS before and after 4 weeks of a LFM diet (60) (day 0 and day 28). IBS-SSS includes 5 questions of equal weight concerning symptoms over the past 10 days, and each question is scored on a 0–100 scale. The scores for all 5 questions are summed to a total IBS-SSS score between 0 and 500, with higher scores suggesting higher symptom severity. IBS-SSS is responsive to treatment, and a ≥ 50-point decrease in IBS-SSS is considered indicative of a responder (60, 61). PROMIS scales of Belly pain and diarrhea were administered to assess the severity of belly pain and diarrhea, and they were administered before and after LFM diet (day 0 and 28) (62). The PROMIS belly pain questionnaire and PROMIS diarrhea questionnaire have 5 and 6 questions, respectively; both questionnaires assess symptom severity on a 5-point Likert scale. Higher T-scores on these questionnaires refer to more severe gastrointestinal symptoms (62). In addition, patients were asked to report the most common stool consistency using BSFS and stool frequency every day during the screening period, and they underwent 4 weeks of dietary intervention; the average of daily stool consistency and frequency during day –7 to 0 was taken as the baseline, and the average during day 21 to 28 was taken as the post-LFM value.

### Selection criteria for human studies.

Adult patients with IBS-D (aged 18–65 years) who met the ROME IV criteria (3) for IBS-D were recruited if they did not have any alarm features (rectal bleeding, weight loss, nocturnal symptoms, family history of inflammatory bowel disease, or celiac disease). In addition, they met the following inclusion criteria (a) normal serum studies, including serum tissue-transglutaminase antibodies, thyroid stimulating hormone levels, C-reactive protein, and complete blood count since the onset of symptoms; (b) normal stool studies, including ova and parasites since the onset of symptoms; and (c) a IBS-SSS score of ≥ 175 at the end of the 7-day screening period.

Patients were excluded from the study if they met any of the following exclusion criteria: they were already on a LFM diet or other dietary restriction such as gluten-free or lactose-free diet within the past 6 months; (b) they had any known food allergy or insulin-dependent diabetes; (c) they had known history of celiac disease, inflammatory bowel disease, or microscopic colitis; (d) they had prior small bowel or colonic surgery or cholecystectomy; (e) they were pregnant; (f) they were on antibiotics in the past 3 months; or (g) they regularly used mast cell stabilizers or antihistaminic or nonsteroidal antiinflammatory agents or steroids, or bile acid binders.

### Barrier function and mast cell activation in patients with IBS-D.

Colonic tight junction protein gene expression was assessed using colon biopsies obtained from patients with IBS-D before and after 4 weeks of a LFM diet. In addition, serum levels of mast cell mediators (tryptase and histamine) were measured before and after LFM diet. ELISA for tryptase and histamine were performed using human wide-range tryptase ELISA (WEB070Hu, American Research Products Inc.) and histamine EIA kit (589651, Cayman Chemical) according to the manufacturer’s instructions.

### Fecal supernatant preparation and LPS measurement.

Fecal samples were collected from patients with IBS-D before and after 4 weeks of a LFM diet stored at –80°C. Based on our recent studies, fecal samples were diluted (1 g fecal sample/5 mL PBS), homogenized on ice, and centrifuged (10,000*g*, 10 minutes, 4°C). The supernatants were recovered, filtered on 0.22 μm filters to remove bacteria, and then stored at –80°C. LPS levels were measured with a quantitative chromogenic limulus amoebocyte lysate (LAL) QCL-1000 test kit (Lonza), following the manufacturer’s protocols.

### In vivo experiments with fecal supernatants.

In total, 200 μL fecal supernatants from patients with IBS-D (before and after LFM) from the same patients described above were administered intracolonically to naive WT mice for 5 days. On day 6, in vivo FITC-dextran measurement and TEER were measured as described above.

### In vitro experiments with fecal supernatants.

BMMCs were obtained from male C57BL/6 mice. Mice were sacrificed, and femurs and tibias were isolated. The bones were flushed with PBS to remove BM, which was cultured for 6–8 weeks in RPMI 1640 medium supplemented with 10% FBS, 1 mM pyruvate, 20 ng/mL IL-3, 100 U/mL penicillin, and 0.1 mg/mL streptomycin as reported (all from Thermo Fisher Scientific) (63). The number of BMMCs was counted by a phase-contrast microscope and 1 × 10^5^ BMMCs per well will be plated in 24-well plates. BMMCs derived from WT mice were stimulated with 30 μL fecal supernatants (from healthy controls and patients with IBS-D before and after 4 weeks of a LFM diet). To study if the effect of IBS fecal supernatant on mast cells were mediated via the LPS/TLR4 pathway, the effect of IBS-D supernatants (pre-LFM) were also compared between BMMCs derived from WT mice and *tlr4*^–/–^ mice. The *tlr4*^–/–^ mast cells were not treated with post-LFM fecal supernatants.

### Statistics.

Differences between HFM and RC groups were compared by 2-tailed unpaired Student’s *t* test. Differences between patients with IBS-D before and after LFM were compared using 2-tailed paired *t* test. When more than 2 groups were compared, 1-way ANOVA followed by Dunnett’s multiple-comparison test was performed. For ANOVA, *P* values in the results section show the results of adjustment for multiple comparisons. Results are expressed as the mean ± SEM. *P* less than 0.05 was considered statistically significant.

### Study approval.

All experimental procedures were performed in accordance with NIH guidelines and approved by the University Committee on Use and Care of Animals at the University of Michigan (approval no. PRO00008525). Clinical study was approved by the University of Michigan IRB (HUM00166423). Informed written consent was obtained from all patients prior to the inclusion in the study.

## Author contributions

CO and PS conceived and designed the study. PS, GG, SYZ, YZ, and JG acquired, analyzed, and interpreted the data. PS and CO wrote the manuscript.

## Supplementary Material

Supplemental data

## Version 1 10/07/2021

In-Press Preview

## Version 2 11/22/2021

Electronic publication
