# Supplementary material for: High FODMAP diet causes barrier loss via lipopolysaccharide-mediated mast cell activation
Source: JCI Insight. 2021 Nov 22;6(22):e146529. doi: 10.1172/jci.insight.146529 (PMC8663790; doi:10.1172/jci.insight.146529)

Supplemental figure 1: Effect of high FODMAP (HFM) diet on tight junction and cytokime mRNA expression in wild type mice: Adult male C57BL/6 mice were randomized for 2 weeks to an HFM diet vs. regular chow (RC). **(A)** HFM diet caused no significant decrease in relative mRNA expression of tight junction proteins (JAM-A and Claudin-1) and **B)** no significant difference in mRNA expression of inflammatory markers (IL-1β, IL-10, and TNF-α (n=8/group). P<0.05 for each using unpaired t-test

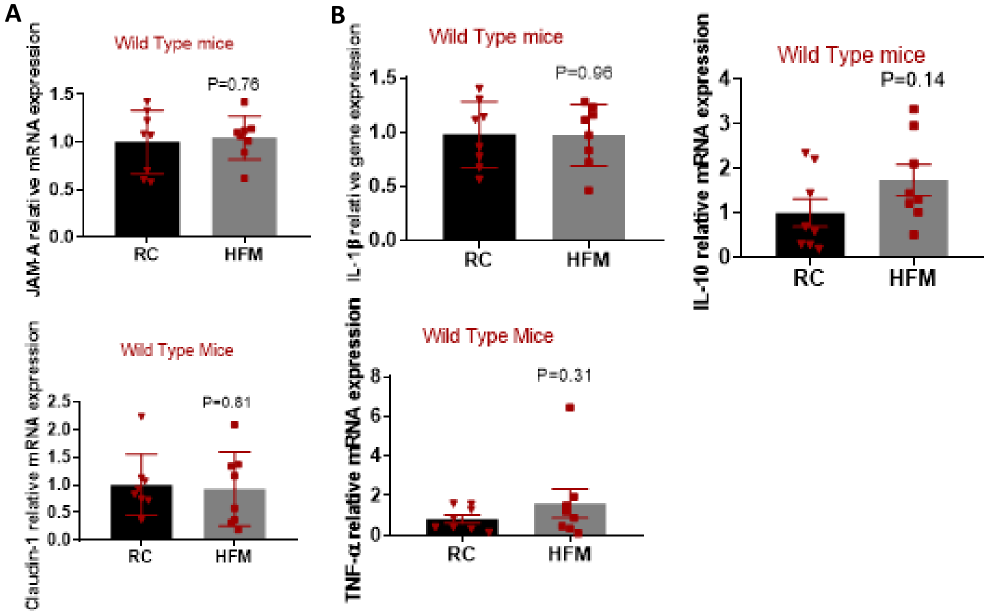

Supplemental figure 1: Effect of high FODMAP (HFM) diet on tight junction and cytokime mRNA expression in mast cell-deficient (MCD) mice: Adult male Mast cell deficient Kit$^{W/W-v}$ (MCD) mice were randomized for 2 weeks to an HFM diet vs. regular chow (RC). **(A)** HFM diet caused no significant decrease in relative mRNA expression of tight junction proteins (ZO-1, Occludin, JAM-A and Claudin-1) and **B)** no significant difference in mRNA expression of inflammatory markers (IL-1β, TNF-α, IFN-γ, IL-4, IL-6, IL-10, and IL-17(n=4/group). P<0.05 for each using unpaired t-test

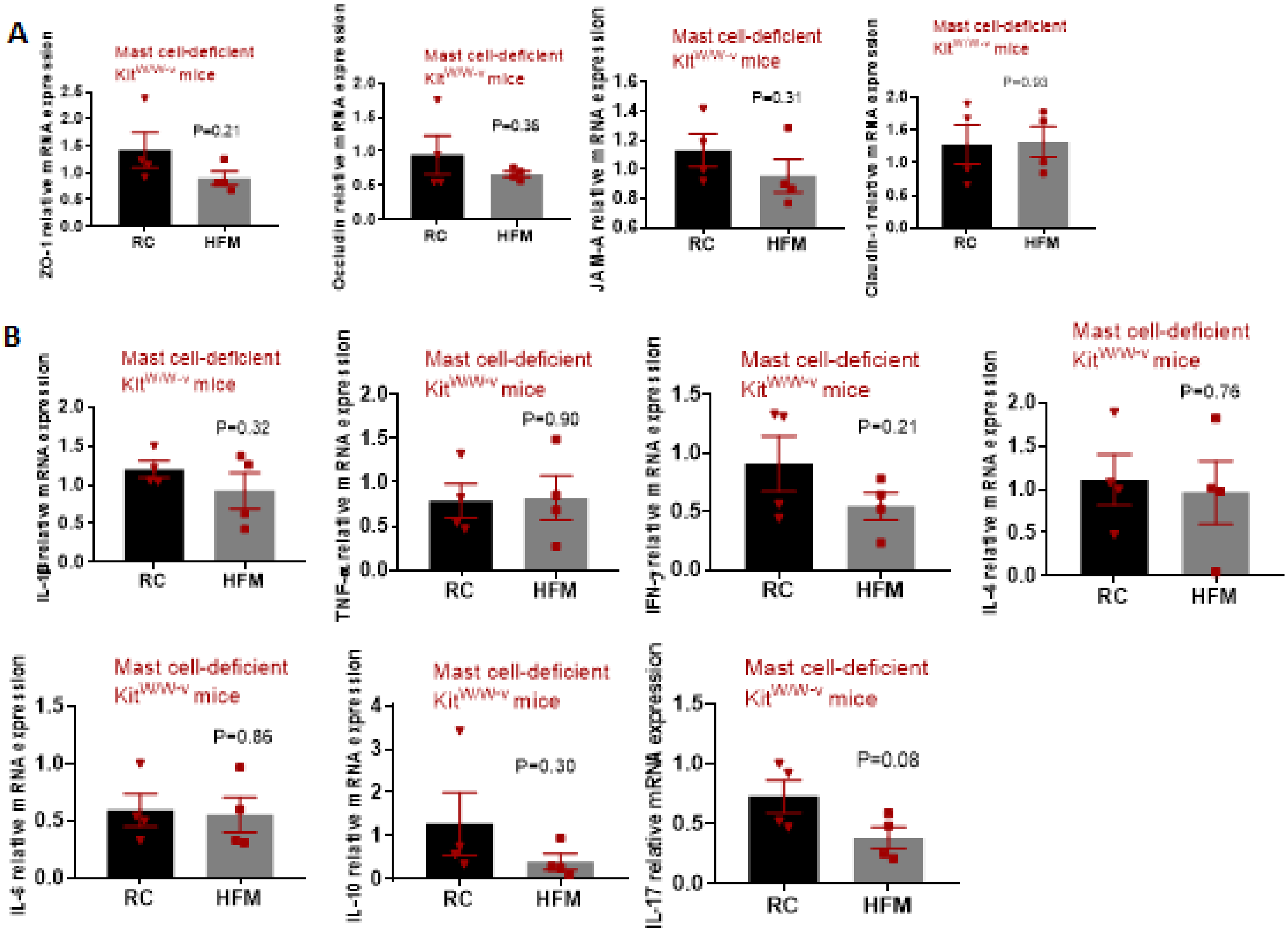

Supplement: Supplemental data [file jciinsight-6-146529-s158.pdf]
